# 959. It Takes a Village: Reducing Empiric Vancomycin Use by Leveraging Primary Team Pharmacist Oversight of a 72-hour Approval Process

**DOI:** 10.1093/ofid/ofac492.802

**Published:** 2022-12-15

**Authors:** Natasha N Pettit, Cynthia T Nguyen, Alison K Lew, Jennifer Pisano

**Affiliations:** University of Chicago Medicine, Chicago, Illinois; University of Chicago Medicine, Chicago, Illinois; University of Chicago Medicine, Chicago, Illinois; University of Chicago Hospital, Chicago, Illinois

## Abstract

**Background:**

Vancomycin is often initiated empirically in hospitalized patients for broad spectrum gram-positive coverage, however in many cases it is initiated unnecessarily and/or continued empirically for longer durations than necessary. Strategies to facilitate timely discontinuation of vancomycin when unnecessary may reduce antibiotic toxicity (e.g. nephrotoxicity) and the development of bacterial resistance. In large hospital systems, it may not be feasible for stewardship programs to implement prior authorization requirements or prospective audit/feedback for all vancomycin orders. Novel strategies to enlist other clinicians to serve as stewards of antibiotic use are needed.

**Figure:**
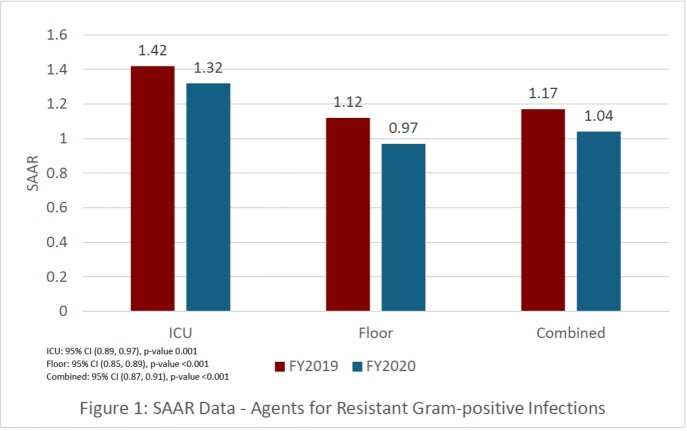

**Methods:**

On February 1, 2020, we implemented a protocol requiring providers to obtain approval from the primary team pharmacist to continue empiric vancomycin regimens >72 hours. Primary team pharmacists were provided a list of appropriate indications for vancomycin continuation. After 72 hours, the pharmacist placed a note describing the approved indication or if ASP or ID consult was obtained. We evaluated the standardized antibiotic administration ratio (SAAR) for antibacterial agents for resistant gram-positive infections for FY2019 (pre-protocol) and FY2020 (post-protocol) to assess the impact of this intervention. Vancomycin utilization was also evaluated in days of therapy (DOT)/1000 patient days during both time periods.

**Results:**

The SAAR for antibacterial agents for resistant gram-positive infections for FY2019 was 1.17 and FY2020 was 1.04 (95% CI (0.87, 0.91), p-value < 0.001). A significant reduction in SAAR was observed in patients admitted to the ICU and floor (Figure 1). Overall vancomycin utilization according to days of therapy/1000 patient days was reduced from 111.13 (FY2019) to 104.08 (FY2020).

**Conclusion:**

Leveraging the oversight of primary team pharmacists proved to be an effective strategy to reduce empiric vancomycin durations of therapy. Following the implementation of a 72-hour approval protocol with primary team pharmacist oversight, we observed a significant reduction in the SAAR for antibacterials for resistant gram-positive infections with a corresponding reduction in vancomycin utilization.

**Disclosures:**

**All Authors**: No reported disclosures.

